# Incident wavelength and polarization dependence of spectral shifts in *β*-Ga_2_O_3_ UV photoluminescence

**DOI:** 10.1038/s41598-018-36676-7

**Published:** 2018-12-24

**Authors:** Yunshan Wang, Peter T. Dickens, Joel B. Varley, Xiaojuan Ni, Emmanuel Lotubai, Samuel Sprawls, Feng Liu, Vincenzo Lordi, Sriram Krishnamoorthy, Steve Blair, Kelvin G. Lynn, Michael Scarpulla, Berardi Sensale-Rodriguez

**Affiliations:** 1 Department of Electrical and Computer Engineering, The University of Utah, Salt Lake City, UT 84112 USA; 2 Department of Mechanical and Materials Engineering, Washington State University, Pullman, WA 99164 USA; 3 Department of Materials Science and Engineering, The University of Utah, Salt Lake City, UT 84112 USA; 4 Quantum Simulations Group, Materials Science Division, Lawrence Livermore National Laboratory, Livermore, CA 94550 USA

## Abstract

We report polarization dependent photoluminescence studies on unintentionally-, Mg-, and Ca-doped *β*-Ga_2_O_3_ bulk crystals grown by the Czochralski method. In particular, we observe a wavelength shift of the highest-energy UV emission which is dependent on the pump photon energy and polarization. For 240 nm (5.17 eV) excitation almost no shift of the UV emission is observed between *E*||*b* and *E*||*c*, while a shift of the UV emission centroid is clearly observed for 266 nm (4.66 eV), a photon energy lying between the band absorption onsets for the two polarizations. These results are consistent with UV emission originating from transitions between conduction band electrons and two differentially-populated self-trapped hole (STH) states. Calcuations based on hybrid and self-interaction-corrected density functional theories further validate that the polarization dependence is consistent with the relative stability of two STHs. This observation implies that the STHs form primarily at the oxygen atoms involved in the original photon absorption event, thus providing the connection between incident polarization and emission wavelength. The data imposes a lower bound on the energy separation between the self-trapped hole states of ~70–160 meV, which is supported by the calculations.

## Introduction

Monoclinic *β*-Ga_2_O_3_ is an ultra-wide bandgap oxide semiconductor exhibiting optical absorption onsets at 4.6–4.9 eV depending on incident light direction and polarization^1^. Thus, it is of interest for optoelectronic applications such as UV-transparent electrodes and visible-blind photodetectors^2,3^. The thermodynamic bandgap controlling intrinsic carrier density has not been determined accurately; thus 4.6 eV represents an upper bound estimate. Nonetheless, the fact that its bandgap greatly exceeds those of GaN and SiC makes *β*-Ga_2_O_3_ attractive for the next generation of high-voltage power devices. Its estimated Baliga’s figure of merit is very high^4^ and many advances have been made in terms of doping and contacting using conventional methods. An important advantage of *β*-Ga_2_O_3_ compared to SiC or GaN is that large area native substrates with low dislocation density can be grown by liquid-phase bulk crystal growth methods at relatively low cost.

*β*-Ga_2_O_3_ exhibits complex optoelectronic behavior as a result of its low-symmetry crystal structure, coincidences of energy levels, self-trapped polaron, and excitonic effects^5–7^. Three important features have been reported in the literature: (*i*) its optical properties are highly anisotropic, (*ii*) the minimum direct optical transition is symmetry-forbidden at its lowest energy, and (*iii*) its emission spectrum does not typically exhibit near-band-edge emission^8^. The optical absorption onset of *β*-Ga_2_O_3_ is known to be a strong function of linear polarization and light incidence orientations^9–13^, which can be understood on the basis of a suppression of the transition matrix elements of its three top valence bands at the *Γ* point when *E*||*b*. Furthermore, (*iv*) its luminescence spectrum is typically composed of four emission bands at ultraviolet (UV and UV’ at approximately 350 and 400 nm respectively), blue (B at approximately 450 nm), and green (G at approximately 500 nm) wavelengths. Whereas the UV’, blue, and green luminescence are attributed to transitions involving deep donors and acceptors^14,15^ as well as self-trapped excitons, the UV luminescence has been attributed to recombination of free electrons and self-trapped holes (STHs) on the two crystallographically-distinct trigonally-coordinated oxygens in the unit cell^16^.

Despite the growing research interest in *β*-Ga_2_O_3_, only a handful of studies report on the effect of extrinsic dopants on the optical properties of single crystal *β*-Ga_2_O_3_. Shimamura *et al*.^17^ reported that Si dopants are effective donors which red-shift the absorption onset and decrease the total luminescence while increasing the relative UV/blue intensity. Mg is expected to behave as an acceptor^18^ and Onuma *et al*.^19^ reported increases in blue and green cathodoluminescence (CL) with Mg doping. In this work, we report studies of the photoluminescence (PL) emission from unintentionally-doped (UID) and acceptor-doped (Mg and Ca) *β*-Ga_2_O_3_ bulk crystals, including dependencies on incident linear polarization. Our studies further elucidate the complexities of the optical processes in *β*-Ga_2_O_3_ and begin to illuminate the role of group-II acceptors such as Mg and Ca.

## Results and Discussion

We used the Czochralski method to grow a series of *β*-Ga_2_O_3_ crystals using CO_2_ to dynamically control the O_2_ partial pressure (pO_2_)^20^. 5N-grade Ga_2_O_3_ powder (GFI Advanced Technologies) was employed as the source material. Glow discharge mass spectrometry (GDMS) analysis of the seed material was consistent with reports in ref. ^21^. Four different melt compositions were employed: unintentionally-doped (UID), Ca-doped 0.20% in the melt, and 0.15% and 0.25% Mg doped in the melt (relative to Ga). The average Mg concentration for the crystal grown for 0.25% doping is expected to be ~0.03% (this is close to the value measured from SIMS, 10^18^ cm^−3^)^22^ with variation of less than 0.002% between the top and bottom. Due to Mg doping being far below 0.15% (an upper bound on the solubility of Mg in *β*-Ga_2_O_3_), no second phase formation is expected, and none was observed in the crystals grown. Despite the different nominal doping of the melts used to grow the crystals, their electrical conductivities were very similar, including for Mg doping which has been reported to result in high resistivity through compensation. The unintentionally-doped and 0.15% Mg samples were grown with an atmosphere of 5000 ppm O_2_ (balance 1:1 N_2_ and CO_2_). The Ca-doped sample was grown in pure CO_2_, while the 0.25% Mg sample was grown with 100 ppm O_2_ (balance N_2_) and ~2% O_2_ (balance N_2_). Thus, we expect that the concentration of O vacancies varies between the samples in addition to the intentional Mg or Ca doping. Therefore, in this work we do not attempt to interpret the PL emission bands believed to involve point defect transitions and focus only the UV spectral features believed to be related to the band structure of the *β*-Ga_2_O_3_ lattice.

**Figure 1:**
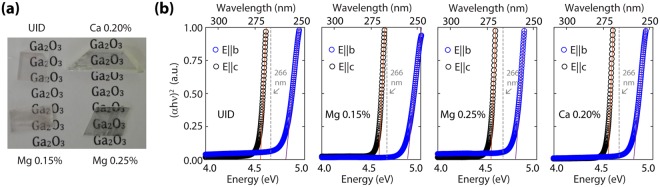
(**a**) Photograph of the analyzed samples. (**b**) Absorption spectra for the analyzed samples. Measurements were carried out in flakes exfoliated from the crystals pictured in (**a**).

All crystals had high clarity with minimal to no discernable color, as opposed to many literature reports. Energy-dispersive spectroscopy (not shown) revealed the presence of Ga and O at the right stoichiometry. Depicted in Fig. 1(a) is an optical image of the crystal samples after dicing. The crystals cleave easily and we verified using X-ray diffraction (Supplementary Fig. S1) that the cleavage planes are {100} using the conventional unit cell defined with *a* = 12.23 Å, *b* = 3.04 Å, *c* = 5.80 Å, and β = 103.83°. Samples showed some reflective features associated with the saw-cut edges which we believe to be partially cleaved cracks on {100} planes that may have formed during crystal cooling. Thus, to try to avoid possible artifacts from cracks having different composition than bulk, we cleaved thin flakes from the pictured samples. For all experiments the direction of light incidence was along the a* direction, normal to the cleaved {100} faces.

Characteristic absorption spectra in the UV range (4.0 to 5.0 eV) for exfoliated flakes, each some tens of μm average thickness, are shown in Fig. 1(b). The expected variation of absorption edge with polarization^9^ was observed across all of the analyzed samples. When the incident polarization is parallel to the *b*-axis (*E*||*b*), the absorption edge occurs close to 250 nm while it shifts to ~270 nm for polarization parallel to the *c*-axis (*E*||*c*). No significant differences were observed in the absorption onset edge among the different types of samples.

**Figure 2:**
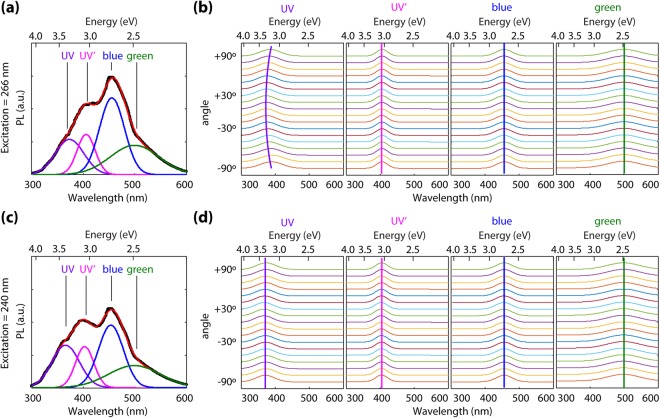
Polarization dependence of photoluminescence. (**a**) PL spectra for the nominally 0.15% Mg-doped sample at 266 nm and its fitting to four Gaussian peaks corresponding to UV, UV’, blue, and green emission. (**b**) Evolution of PL peak positions with excitation polarization angle at 266 nm (polarization 0° is parallel to *c*-axis) (**c**) PL spectra for the nominally 0.15% Mg-doped sample at 240 nm and its fitting to four Gaussian peaks corresponding to UV, UV’, blue, and green emission. (**d**) Evolution of PL peak positions with excitation polarization angle at 240 nm.

In order to qualitatively compare the relative intensities of emission features from the UID, Ca-doped, and Mg-doped samples as a function of excitation wavelength, their PL spectra are displayed in arbitrary units. At this time, in order to avoid errors related to pump power, we do not make comparisons of absolute intensities of emission between the 240 and 266 nm excitation cases. PL spectra were taken as a function of incident polarization for all samples. The spectra at each polarization angle were fit using nonlinear least square method to four Gaussian peaks associated with the UV, UV’, blue, and green emission bands. The position of the center, amplitude and width of each Gaussian curve were set as fitting parameters. Depicted in Fig. 2(a) is the PL spectrum for the nominally 0.15% Mg-doped sample excited at 266 nm with incident polarization parallel to the *c*-axis, together with its best fit. By fitting the spectra at different polarization angles, the plots depicted in Fig. 2(b) are obtained, which represent the center wavelength of each of the four Gaussian peaks. Polarization angle 0 degrees is parallel to the *c*-axis, and 90 degrees is parallel to the *b*-axis. The same procedure was followed employing 240 nm excitation and the results are depicted in Fig. 2(c,d). As seen in Fig. 2(b) at 266 nm (4.66 eV), the UV (~350 nm) peak shifts its center with polarization angle, especially for the Ca and Mg doped samples. This is a remarkable finding that indicates “memory” of the incident polarization, despite thermalization of generated carriers to the band edges. This is contrary to our observations for 240 nm pump wavelength (5.17 eV, where no anisotropy in absorption exists) depicted in Fig. 2(d), where negligible shift in the position of the UV peak is observed. In semiconductors, PL polarized according to the same linear polarization as the excitation light is not unprecedented – defect related emission in diamond and other materials was first reported many decades ago. However, in such defect emission cases the PL was excited at sub-bandgap energies with absorption directly into the emitting defects. Thus, despite the defects existing for all symmetry-identical orientations in the samples, subpopulations of the defects for which the dipole moment aligned along the polarization axis were selectively excited thus leading to linearly polarized emission^23–26^. The fact that only very weak PL barely above background is observed for sub-bandgap excitation in the current case of *β*-Ga_2_O_3_ rules out this mechanism.

Rather, in the current *β*-Ga_2_O_3_ experiments we hypothesize that the observed spectral shifts with incident wavelength and polarization can be explained in terms of emission transitions involving crystallographically-distinct self-trapped holes. As identified previously^6,16,27^, our calculations show that holes exhibit a strong preference to self-trap and form small polarons on O sites. Relative to a free hole, we compute the self-trapping energies to be –0.39 eV for holes localized on O_I_ atoms and –0.46 eV for holes localized on O_II_ atoms. We find the wave functions associated with the small polarons formed on the trigonal O_I_ and O_II_ sites are consistent with previous reports^6,16^ with the O p-orbitals involved highly oriented parallel to the *a*-axis for the O_I_ site and parallel to the *c*-axis for O_II_, which ultimately leads to the polarization dependence observed.

**Figure 3:**
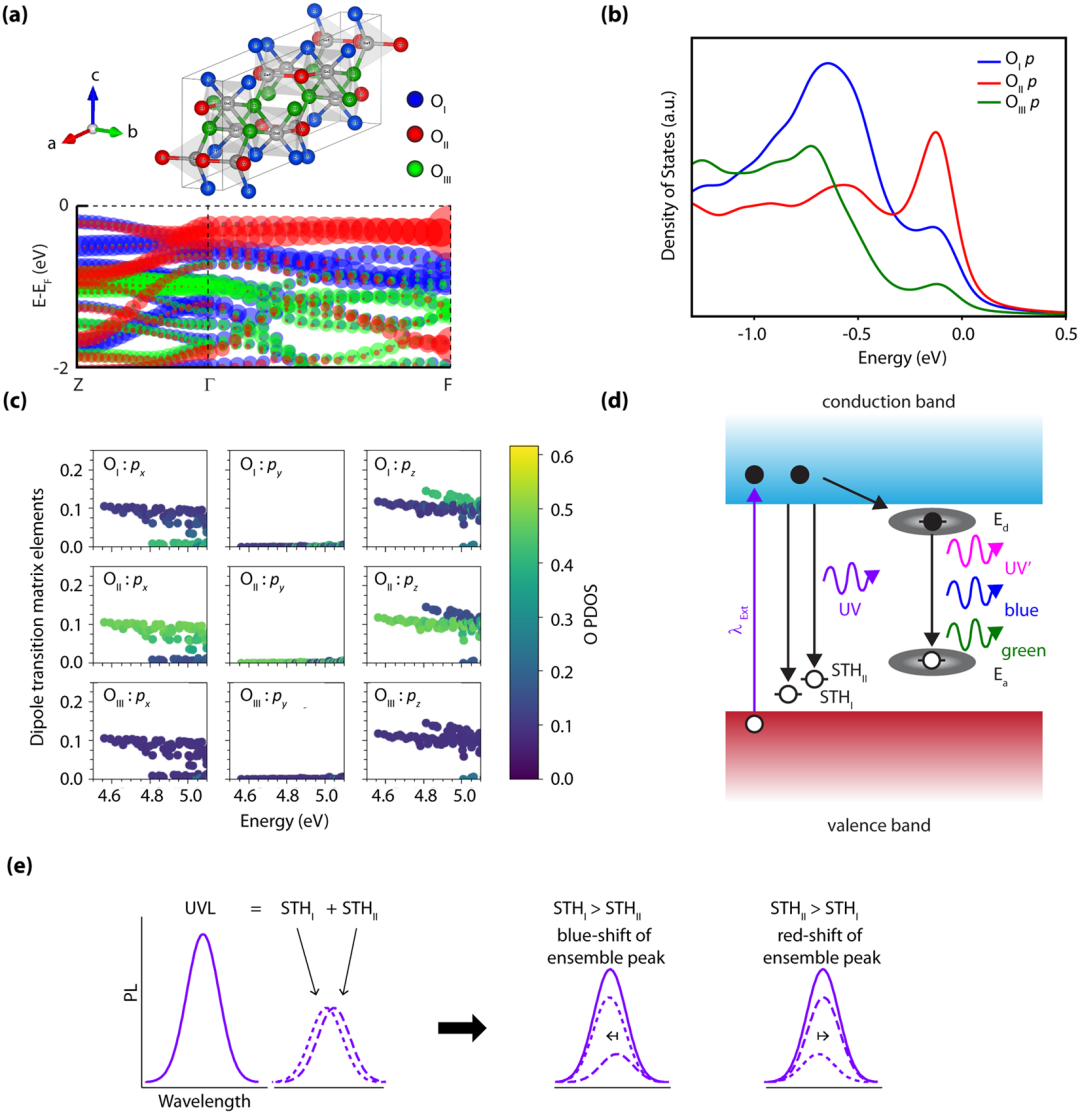
Theoretical analysis of the valence band states as an explanation for the observed UV emission wavelength shift under 266 nm excitation. (**a**) Computed *β*-Ga_2_O_3_ valence band structure along the *k*-path: Z = (0.0 0.0 0.5) − Γ = (0.0 0.0 0.0) − F = (0.0 0.5 0.0), where the points are colored terms of O_I_, O_II_ and O_III_ contributions with blue, red, and green, respectively. (**b**) Projected density of states (PDOS) showing the O *p*-orbital contributions to the valence band for the three distinct crystallographic O sites. The calculated data is broadened with Gaussian and Lorentzians with a full-width half maximum of 0.10 eV. (**c**) Analysis of the dipole transition matrix elements from valence bands to the lowest-lying conduction band for each crystallographic direction, showing the relative contributions of each O site as a heatmap. All of the plots suggest that O_II_ sites contribute the most to absorption processes involving the highest-lying valence bands. (**d**) Illustration of potential emission paths leading to the observed PL peaks based on the calculate polaron single-particle states. (**e**) Explanation of the observed UV PL wavelength shift on-basis of the observed peak being the ensemble of two distinct Gaussians corresponding to two distinct STH levels associated with the O_I_ and O_II_ sites.

In Fig. 3(a), we show the computed character of the valence band states associated with each of the crystallographically distinct O sites, in reciprocal space parallel to the *b* and *c* axes. Figure 3(b) shows the projected density of states (PDOS) of the 2p orbitals for each O site, along with the associated optical dipole transition matrix elements between the valence states and the lowest conduction band states over a densely-sampled mesh including 3614 *k*-points in Fig. 3(c). The PDOS in Fig. 3(b) clearly show that all three O sites contribute to the uppermost valence bands, but the dominant contribution at the band edge is from O_II_ sites. The O_I_ site has the strongest contribution to states ~0.5–1 eV below the VBM. Both the O_I_ and O_II_ states can contribute to absorption at the near above-band gap wavelength used in this work. This is supported by the band structure analysis in Fig. 3(a), where the top two valence bands consist of states from the trigonal O_I_ and O_II_ atoms. The contributions of the tetragonally-coordinated O_III_ atoms are limited to a band significantly lower in energy and thus will not participate in optical absorption except for excitation energies well above the absorption onset (i.e. photon energies well above those used in this work).

The calculated optical dipole transition matrix elements in Fig. 3(c) are shown decomposed by O site and p-orbital orientation, as labeled. The results show the clear anisotropy of the absorption edge, with a lower-energy band associated with polarization parallel to the *c*-axis (O_II_ p_x_, p_z_) and weak absorption for polarization parallel to the *b*-axis (O_I_ p_y_), as reported previously^12,13^. For higher energies, a strong absorption band associated with O_I_ p_z_ is observed. The results in Fig. 3 suggest that for 266 nm (4.66 eV) excitation just above the absorption threshold, E||b absorption can only occur involving the highest energy valence bands that are primarily derived from O_II_ states; whereas, when E||c both O states may more equally contribute to absorption. For 240 nm (5.17 eV) excitation corresponding to photon energy sufficiently high that absorption can occur over a wider range of directions and *k* values, both O_I_ and O_II_ states can strongly participate in absorption regardless of the incident light polarization. Therefore: (i) the populations of holes created by photo-absorption will be associated mainly with O_II_ atoms for 266 nm excitation (E||b) while holes will be created at both O_I_ and O_II_ atoms for 266 nm (E||c) and 240 nm excitations. (ii) At 266 nm excitation, the ratio of holes excited at O_I_ and O_II_ sites can be modified by incident polarization. These two effects combine to produce the wavelength and polarization dependence of populations of holes generated at O_I_ and O_II_ positions in the lattice.

In the limit that self-trapped holes form at the site of the hole created by photo-absorption before hopping to other sites, we thus expect STHs to form according to the populations of photoexcited holes. To validate this assumption, we evaluated the barrier for polaron hopping between the O_II_ and O_I_ sites using the climbing image nudged elastic band method within the pSIC + PBE-GGA formalism^28,29^. We calculate a barrier of 0.14 eV to hop from the more stable O_II_ polaron configuration to O_I_, which corresponds to an average hopping time at room temperature ~12–60 ps, assuming a range of attempt frequencies given by the optical phonon modes in Ga_2_O_3_^30^. Since the polaron hopping time is significantly longer than the sub-10 ps radiative lifetime measured with time-resolved photoluminescence at room temperature^27^, the STHs do not have time to change sites after formation from the initial absorption event at room temperature. This condition on the dynamics of recombination and transport, combined with the polarization-dependent absorption, thus provides a mechanism by which the crystal can “remember” the incident polarization, which is reflected in the emission. The observed centroid shift of the UV emission requires one additional condition: that the STH levels associated with O_I_ and O_II_ are non-degenerate in energy. The pSIC calculations predict the polaron states to lie at 1.02 and 1.10 eV above the VBM for the O_I_ and O_II_ STH, respectively, thus giving rise to photoluminescence emission shifted upward by 80 meV for recombination by a conduction band-edge electron with an O_I_-excited STH. Thus, in our interpretation the UV emission peak actually consists of two closely-spaced peaks corresponding to CB→STH_I_ and CB→STH_II_ transitions as illustrated in Fig. 3(d). The relative intensity of these two components making up the UV peak would thus vary with incident wavelength and polarization leading to a wavelength shift in the centroid of the ensemble peak as illustrated Fig. 3(e). The observed wavelength shift represents a lower bound on the magnitude of the separation between the STH levels; maximal spectral shifting would be observed for the case where the STH_I_ and STH_II_ states varied from exclusively one to exclusively the other state. The magnitude of the energy shift observed in these samples and our lower-bound estimate for the separation of the STH states is ~70–160 meV, which is in good agreement with the 80 meV predicted from theory.

**Figure 4:**
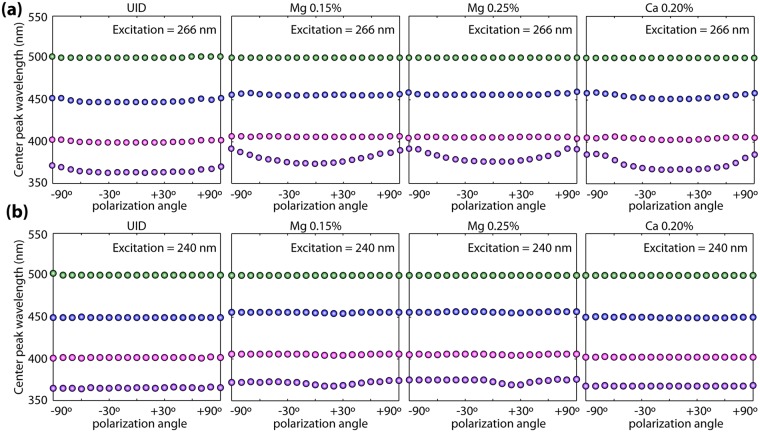
Summary of polarization dependence of PL across all the analyzed samples. (**a**) Evolution of PL peak positions with excitation polarization angle at 266 nm for all the analyzed samples. (**b**) Evolution of PL peak positions with excitation polarization angle at 240 nm for all the analyzed samples.

Summarized in Fig. 4 is the evolution of the UV-ensemble emission wavelength with polarization across all samples. Similar to what has been observed in the nominally 0.15% Mg sample (Fig. 2(b–d)), only negligible variations in peak position are observed for 240 nm excitation across all samples. However, at 266 nm excitation, significant shifts of the UV emission peak are observed for all samples. The independence of this behavior on the Mg and Ca doping and growth atmosphere argues for the origin of the UV peak in the electronic structure of the *β*-Ga_2_O_3_ crystal. It is worth mentioning however that the intentionally doped samples show stronger UV PL wavelength shifts than the UID ones although the origins of this observation are unclear at this point and will be the subject of further studies. We note that holes preferentially bind to the O_I_ atoms adjacent to Mg and Ca dopants, which strongly prefer to incorporate on the octahedral Ga sites^22,31^. The extent to which this is responsible for the slight variations in the signals of the O_I_ and O_II_ luminescence signals between the UID and doped samples in Fig. 4 will be investigated in future work.

## Conclusions

In conclusion, we reported polarization dependent PL studies on unintentionally-, Mg-, and Ca-doped *β*-Ga_2_O_3_ bulk crystals. In particular, we observe wavelength shifts of the highest energy UV emission under 266 nm excitation; a phenomenon that was not observed at 240 nm excitation. These observations are consistent with theoretical studies of the polarons that the emission originates from transitions between conduction band electrons and two differentially-populated STH states on the O_I_ and O_II_ sites of the *β*-Ga_2_O_3_ lattice. The measured magnitude of the energy shift observed in these samples was ~70–160 meV and in good agreement with the calculated value of 80 meV, with the O_II_ polaron leading to a slightly red-shifted luminescence relative to the O_I_ polaron.

## Methods

For *absorption measurements*, a high-brightness fiber-coupled broadband light source (LDLSTM Laser-Driven Light Source Model EQ-99-FC) was employed, providing wavelengths from the mid-UV through the visible. Light was initially linearly-polarized using a Rochon prism and passed through polarization rotation optics, with final linear polarization lying in the (100) plane defined by the *b* = [010] and *c*** = **[001] lattice vectors. The transmitted light was collected by a lens-coupled optical fiber connected to an AvaSpec-USB2-DT spectrometer with detection range from 350 to 800 nm. The intensity spectrum measured without a sample was used as the reference signal (thus the data was not corrected for reflections).

*Photoluminescence* was excited by ultrafast (fs) pulses from a wavelength-tunable Ti:Sapphire laser passed through a third-harmonic generator. Three excitation wavelengths were chosen: 240 nm (5.17 eV), which is sufficiently high in energy that no anisotropy is observed in transmission; 266 nm (4.66 eV), which is in the energy range where maximum absorption anisotropy is observed; and 273 nm (4.54 eV), which is below the detectable absorption threshold for all incident polarizations in the *b*-*c* plane. No data is shown for 273 nm excitation because only very weak PL was detected for all samples at this pump wavelength. The PL spectra were collected at room temperature within a 3.75” integrating sphere with the sample at the exit port of the sphere (to avoid collection of transmitted excitation light) and the fiber from the AvaSpec spectrometer inserted into a port opposite the sample but at an oblique angle (to avoid collection of reflected excitation light). Thus, the PL emission was neither resolved in terms of its emission direction nor its polarization.

*First-principles calculations* of the electronic structure of the bulk and polaron configurations were performed using the Vienna ab initio simulation package (VASP)^32^. Atom-projected band structure, density of states (DOS), and dipole transition matrix elements were evaluated using the Perdew-Burke-Ernzerhof generalized gradient approximation functional (PBE-GGA) and a plane-wave cutoff of 400 eV. The DOS and dipole matrix elements were evaluated using a 24 × 24 × 24 Γ-centered k-point mesh. Polarons were modeled using 160-atom supercells and the recently developed variational polaron self-interaction-corrected total energy (pSIC) functional^28^, which was shown to properly capture the effects of polaronic charge localization. Self-consistent lattice parameters were used within each level of theory^6^.

For the determination of polaronic energy levels of the STHs, the pSIC functional has the advantage of working with neutral supercells and also being insensitive to the starting level of theory (e.g., PBE vs. hybrid)^27^, as opposed to other approaches using adjustable parameters, such as DFT + U or variable-mixing hybrid functionals. We confirmed that the predictions of the STH energy levels were within 0.04 eV for both PBE-GGA and PBE-based hybrid functional using 32% mixing of exact exchange, despite the much more accurate band gap prediction using the latter level of theory^6^. In addition, the use of neutral cells with the pSIC methodology eliminates the need to correct spurious periodic image-charge interactions, which otherwise add uncertainty to the calculations^16^.

## Electronic supplementary material

Supplementary Information

## References

[1] Peelaers H, Van de Walle CG (2015). Brillouin zone and band structure of *β*-Ga_2_O_3_. Phys Status Solidi B.

[2] Ji Z, Du J, Fan J, Wang W (2006). Gallium oxide films for filter and solar-blind UV detector. Optical Materials.

[3] Mahmoud WE (2016). Solar blind avalanche photodetector based on the cation exchange growth of *β*-Ga_2_O_3_/SnO_2_ bilayer heterostructure thin film. Solar Energy Materials and Solar Cells.

[4] Higashiwaki M (2016). Recent progress in Ga_2_O_3_ power devices. Semicond. Sci. Technol..

[5] Villora EG (2002). Optical Spectroscopy Study on *β*-Ga_2_O_3_. Jpn. J. Appl. Phys..

[6] Varley JB, Janotti A, Franchini C, Van de Walle CG (2012). Role of self-trapping in luminescence and p-type conductivity of wide-band-gap oxides. Phys. Rev. B.

[7] Tang C (2016). Electronic structure and optical property of metal-doped Ga_2_O_3_: a first principles study. RSC Adv..

[8] Mengle KA, Shi G, Bayerl D, Kioupakis E (2016). First-principles calculations of the near-edge optical properties of *β*-Ga_2_O_3_. Appl. Phys. Lett..

[9] Yamaga M (2011). Polarization of optical spectra in transparent conductive oxide *β*-Ga_2_O_3_. Phys. Status Solidi C.

[10] Yamaguchi K (2004). First principles study on electronic structure of *β*-Ga_2_O_3_. Solid State Communications.

[11] Ricci F (2016). Theoretical and experimental investigation of optical absorption anisotropy in *β*-Ga_2_O_3_. J. Phys.: Condens. Matter.

[12] Onuma T (2015). Valence band ordering in *β*-Ga_2_O_3_ studied by polarized transmittance and reflectance spectroscopy. Jpn. J. Appl. Phys..

[13] Varley JB, Schleife A (2015). Bethe–Salpeter calculation of optical-absorption spectra of In_2_O_3_ and Ga_2_O_3_. Semicond. Sci. Technol..

[14] Ho CH, Tseng CY, Tien LC (2010). Thermoreflectance characterization of β-Ga_2_O_3_ thin-film nanostrips. Opt. Express.

[15] Dong L, Jia R, Xin B, Peng B, Zhang Y (2017). Effects of oxygen vacancies on the structural and optical properties of β-Ga_2_O_3_. Sci. Rep..

[16] Deák P (2017). Choosing the correct hybrid for defect calculations: A case study on intrinsic carrier trapping in *β*-Ga_2_O_3_. Phys. Rev. B.

[17] Shimamura K, Villora EG, Ujiie T, Aoki K (2008). Excitation and photoluminescence of pure and Si-doped *β*-Ga_2_O_3_ single crystals. Appl. Phys. Lett..

[18] Neal AT (2018). Donors and deep acceptors in *β*-Ga_2_O_3_. Appl. Phys. Lett..

[19] Onuma T (2013). Correlation between blue luminescence intensity and resistivity in *β*-Ga_2_O_3_ single crystals. Appl. Phys. Lett..

[20] Galazka Z (2014). On the bulk *β*-Ga_2_O_3_ single crystals grown by the Czochralski method. Journal of Crystal Growth.

[21] Kuramata A (2016). High-quality *β*-Ga_2_O_3_ single crystals grown by edge-defined film-fed growth. Jpn. J. Appl. Phys..

[22] Ritter JR (2018). Compensation and of magnesium acceptors in β-Ga2O3. Appl. Phys. Lett..

[23] Patrick L (1960). Polarization in the luminescence of donor acceptor pairs. Phys. Rev..

[24] Clark CD, Maycraft GW, Mitchell EWJ (1962). Polarization of Luminescence. J Appl Phys.

[25] Collins WC, Crawford JH (1972). J. Polarization of Luminescence in NaCl: Pb^2^ and KCl: Pb^2^. Phys. Rev. B.

[26] Clark CD, Norris CA (1971). Photoluminescence associated with the 1.673, 1.944 and 2.498 eV centres in diamond. J. Phys. C: Solid State Phys..

[27] Yamaoka S, Furukawa Y, Nakayama M (2017). Initial process of photoluminescence dynamics of self-trapped excitons in a β-Ga2O3 single crystal. Phys. Rev. B.

[28] Sadigh B, Erhart P, Åberg D (2015). Variational polaron self-interaction-corrected total-energy functional for charge excitations in insulators. Phys. Rev. B.

[29] Henkelman G, Uberuaga BP, Jónsson H (2000). A climbing image nudged elastic band method for finding saddle points and minimum energy paths. J Chem Phys.

[30] Schubert M (2016). Anisotropy, phonon modes, and free charge carrier parameters in monoclinic β-gallium oxide single crystals. Phys. Rev. B.

[31] Lyons JL (2018). A survey of acceptor dopants for β-Ga2O3. Semicond. Sci. Technol..

[32] Kresse G, Hafner J (1992). Ab. initio molecular dynamics for liquid metals. Phys. Rev. B.

