# Supplementary material for: Incident wavelength and polarization dependence of spectral shifts in β-Ga2O3 UV photoluminescence
Source: Sci Rep. 2018 Dec 24;8:18075. doi: 10.1038/s41598-018-36676-7 (PMC6305385; doi:10.1038/s41598-018-36676-7)
Supplement: Supplementary file 1 — Supplementary Information [file 41598_2018_36676_MOESM1_ESM.docx]

**Supplementary material:**

Incident wavelength and polarization dependence of spectral shifts in *β*-Ga_2_O_3_ UV photoluminescence

Yunshan Wang^1^, Peter T. Dickens^2^, Joel B. Varley^4^, Xiaojuan Ni^3^, Emmanuel Lotubai^1^, Samuel Sprawls^1^, Feng Liu^3^, Vincenzo Lordi^4^, Sriram Krishnamoorthy^1^, Steve Blair^1^, Kelvin G. Lynn^2^, Michael Scarpulla^1,3^*, and Berardi Sensale-Rodriguez^1^*

^1^Department of Electrical and Computer Engineering, The University of Utah, Salt Lake City, UT 84112, USA.

^2^Department of Mechanical and Materials Engineering, Washington State University, Pullman, WA 99164, USA.

^3^Department of Materials Science and Engineering, The University of Utah, Salt Lake City, UT 84112, USA.

^4^Quantum Simulations Group, Materials Science Division, Lawrence Livermore National Laboratory, Livermore, CA 94550, USA.


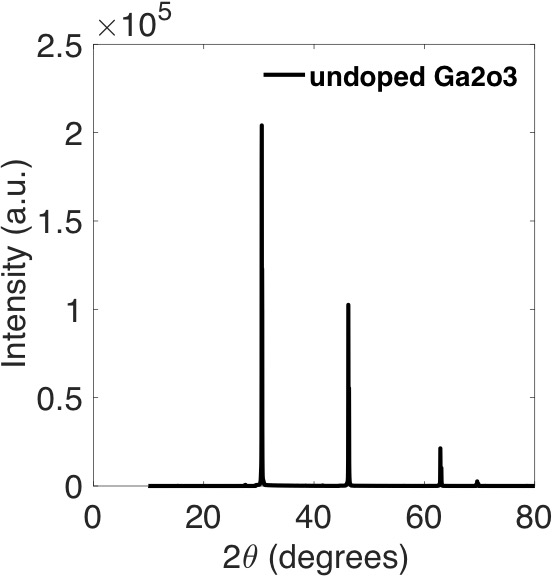


**Supplementary Figure S1:** XRD data of unintentionally doped single crystal *β*-Ga_2_O_3_. Peaks positions at 30 degrees, 46 degrees and 62 degrees correspond to (400), (600), and (800) planes.

**Supplementary Figure S2:** Summary of polarization dependence of PL for the Mg 0.25% sample for 5 different incident wavelengths. Evolution of PL peak position with excitation polarization angle at (a) 240 nm, (b) 243 nm, (c) 246 nm, (d) 253 nm, and (e) 266 nm. The dashed lines correspond to one standard deviation in curve fitting error of UV emission peaks.

**Supplementary Figure S3:** UV peak position at polarizations parallel to b and c axes for the Mg 0.25% sample at different incident photon energies. The error bar represents one standard deviation in curve fitting error of UV emission peaks.
